# On the utilization of polygenic risk scores for therapeutic targeting

**DOI:** 10.1371/journal.pgen.1008060

**Published:** 2019-04-25

**Authors:** Greg Gibson

**Affiliations:** Center for Integrative Genomics and School of Biological Sciences, Georgia Institute of Technology, Atlanta, Georgia, United States of America; Stanford University School of Medicine, UNITED STATES

## Abstract

The promise of personalized genomic medicine is that knowledge of a person’s gene sequences and activity will facilitate more appropriate medical interventions, particularly drug prescriptions, to reduce the burden of disease. Early successes in oncology and pediatrics have affirmed the power of positive diagnosis and are mostly based on detection of one or a few mutations that drive the specific pathology. However, genetically more complex diseases require the development of polygenic risk scores (PRSs) that have variable accuracy. The rarity of events often means that they have necessarily low precision: many called positives are actually not at risk, and only a fraction of cases are prevented by targeted therapy. In some situations, negative prediction may better define the population at low risk. Here, I review five conditions across a broad spectrum of chronic disease (opioid pain medication, hypertension, type 2 diabetes, major depression, and osteoporotic bone fracture), considering in each case how genetic prediction might be used to target drug prescription. This leads to a call for more research designed to evaluate genetic likelihood of response to therapy and a call for evaluation of PRS, not just in terms of sensitivity and specificity but also with respect to potential clinical efficacy.

Much progress has been made in genetic risk assessment for a wide variety of conditions, with implications for implementation of personalized medicine [1]. For the most part, predictions are made with the intention of “positive prediction,” albeit of disease state: the goal is to ascertain who among an at-risk population have the highest likelihood of developing a condition or of progressing to a more severe state. Thus, Kathiresan and colleagues [2] generated genome-wide polygenic risk scores (PRSs) for coronary artery disease, atrial fibrillation, Crohn’s disease, type 2 diabetes, and breast cancer, in each case identifying a threshold above which a small percentage of the population has disease risk at least 3-fold higher than the general population. Because single-gene mutations with such a magnitude of effect are sometimes regarded as clinically actionable, yet affect a much smaller proportion of people, Kathiresan and colleagues argue that PRSs are now at the point at which it is appropriate to integrate them into clinical care. Minimally, this may mean encouraging high-risk individuals to see an appropriate medical specialist or to initiate behavioral change. More commonly, it will engage a course of preventive medication, and as the costs of such medication increase, the impact on both the person and the healthcare system comes into focus. The percentage of incidents prevented by medication will be a function of the proportion of the population who are treated and the rate of response to treatment, which itself may vary, possibly as a function of disease risk.

Recently, I made the argument that because negative prediction is almost always more accurate than positive, owing to the low ratio of cases to controls, the potential for using PRSs to identify low-risk individuals should be given more attention than hitherto [3]. The reason is that if only the highest-risk individuals are treated, then most cases are not prevented, yet treating everyone is both prohibitively expensive (particularly for biologics, which can cost over US$80,000 per patient per year, or in low-GDP countries with developing healthcare infrastructure) and potentially harmful. Both relative and absolute risk can be used to assess efficacy of medications: the former focuses on reducing the rate of incidents (say from 5% to 4%), the latter on reducing the number needed to treat (NNT, say from 50 to 20 incidents prevented for each person taking the drug) [4]. Both of these numbers ought to be considered when approaching the question of how to utilize PRSs in a manner that effectively focuses medical attention on the largest population with a high likelihood of effective response to therapy.

Four variables are critical in making this assessment: the prevalence of the condition, the risk in each PRS-positive target group, the proportion of the population in the group, and the therapeutic response rate. Fig 1 and Table 1 illustrate some of the key relationships among these variables. The top panel (Fig 1A) shows a typical curve of the relationship between prevalence and percentile of genetic risk computed from hundreds of thousands of variants. This allows computation of the precision (percent affected) in each percentile, which is projected below in Fig 1B for conditions with either 2% or 20% overall prevalence (in both cases, following realistic sensitivity as drawn). Table 1 adds data on sensitivity, prevention, and the NNT as a function of effectiveness of the intervention, generated using an online calculator put together to facilitate such analyses: http://accurator.biosci.gatech.edu/. For a rare condition, with realistic relative risk assessments, precision will never exceed 10, but it should be feasible to prevent between one-fifth and one-third of all cases by targeting just the highest-risk 5% to 20% of the population with a highly effective intervention. Prevention of half of the cases would require unrealistically high predictors, at least by current standards. Because the negative predictive values are very high, there is, however, considerable potential utility in recognizing the fraction of individuals unlikely to progress or who will rarely benefit from medication. For a common condition with the same range of relative risk prediction, precision can be better than one in two for the top decile, and most patients will benefit from treatment, resulting in a low NNT. The final columns of Table 1 make the point that, if response rates are considerably higher in the high-risk population, the NNT can be reduced as much as 5-fold, and over half of the preventable events can be avoided by targeting just the high-risk decile.

**Fig 1:**
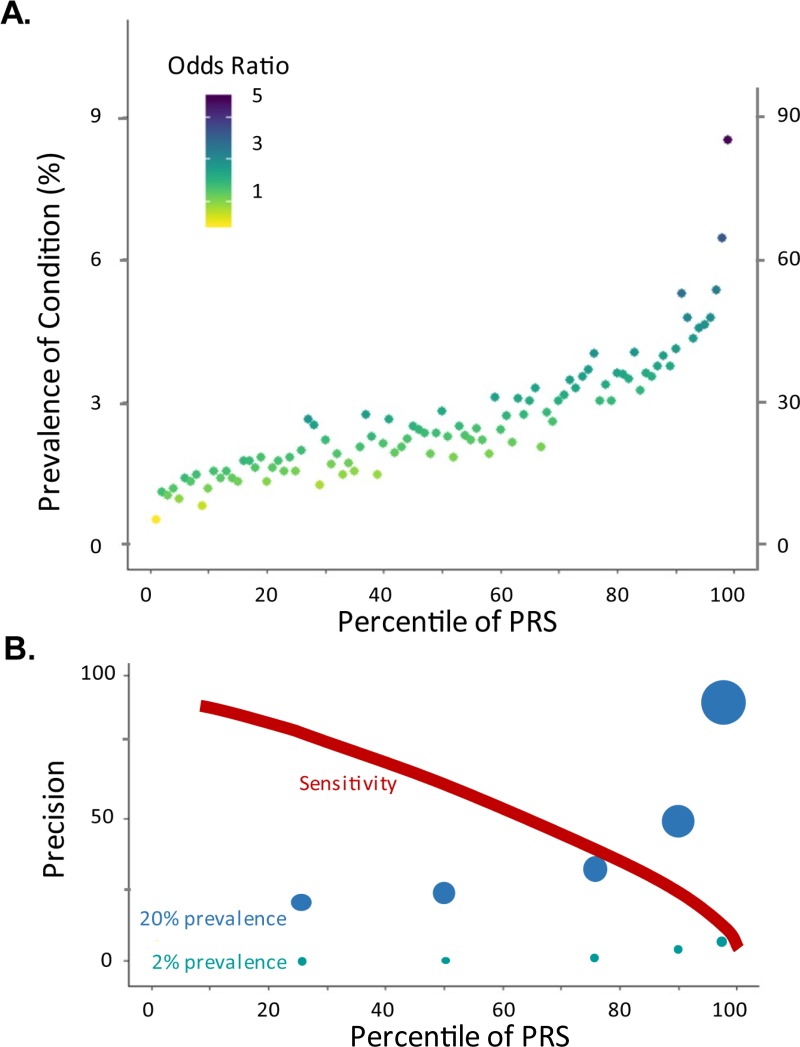
Relationship between PRS, prevalence, and precision. (A) Typical profile of the relationship between percentile of PRS and prevalence of a condition. Each point is estimated from a PRS derived from many thousands of variants in a population of hundreds of thousands of individuals, such as the UK Biobank. The color corresponds to the ratio of prevalence in the indicated percentile to the prevalence in all individuals in lower percentiles. The left axis assumes an overall prevalence of 2%, the right axis 20%. (B) Relationship between Precision and PRS. Precision is the proportion of individuals called positive who actually have the disease, and it is plotted for individuals above the 25th, 50th, 75th, 90th, and 95th percentile for the two prevalances in (A). The diameter of each point is proportional to the indicated Precision, for emphasis. The Sensitivity curve shows the approximate proportion of cases captured by the PRS at the indicated percentile. PRS, polygenic risk score.

**Table 1:** Modeled prevalence, relative risk, response, and the NNT.

Attribute	Rare, Reasonable				Rare, Exceptional				Common, Predictive				Less Effective			
**Prevalence**	**5**	**8**	**8**	**2**	**2**	**2**	**2**	**2**	**20**	**20**	**20**	**20**	**20**	**20**	**20**	**20**
**Relative Risk**	**4.7**	**3.0**	**2.4**	**2.0**	**10**	**9.0**	**5.6**	**4.0**	**4.7**	**3.0**	**2.4**	**2.0**	**4.7**	**4.0**	**4.7**	**3.0**
**Target**	**5%**	**10%**	**15%**	**20%**	**5%**	**10%**	**15%**	**20%**	**5%**	**10%**	**15%**	**20%**	**5%**	**10%**	**5%**	**10%**
**PPV/Precision**	**8**	**5**	**4**	**3**	**14**	**10**	**7**	**5**	**79**	**50**	**40**	**33**	**79**	**62**	**79**	**50**
**NPV**	**98**	**98**	**98**	**98**	**99**	**99**	**99**	**99**	**83**	**83**	**83**	**83**	**83**	**85**	**83**	**83**
**Accuracy**	**94**	**89**	**84**	**79**	**94**	**90**	**85**	**80**	**83**	**80**	**77**	**73**	**83**	**82**	**83**	**80**
**Sensitivity**	**20**	**25**	**30**	**33**	**34**	**50**	**50**	**50**	**20**	**25**	**30**	**50**	**20**	**31**	**20**	**25**
**Specificity**	**95**	**90**	**85**	**80**	**96**	**91**	**86**	**81**	**99**	**94**	**89**	**88**	**99**	**95**	**99**	**94**
**Effectiveness**	**90%**	**90%**	**90%**	**90%**	**90%**	**90%**	**90%**	**90%**	**90%**	**90%**	**90%**	**90%**	**50%**	**50%**	**75/25%**	**75/25%**
**Percent of Preventable**	**20%**	**25%**	**30%**	**33%**	**34%**	**50%**	**50%**	**50%**	**20%**	**25%**	**30%**	**33%**	**20%**	**31%**	**43%**	**50%**
**Percent of All Prevented**	**18%**	**23%**	**27%**	**30%**	**31%**	**45%**	**45%**	**45%**	**18%**	**23%**	**27%**	**30%**	**10%**	**15%**	**15%**	**19%**
**NNT All**	**56**	**56**	**56**	**56**	**56**	**56**	**56**	**56**	**6**	**6**	**6**	**6**	**10**	**10**	**14**	**13**
**NNT Targeted**	**14**	**22**	**28**	**33**	**8**	**11**	**17**	**22**	**1**	**2**	**3**	**3**	**3**	**3**	**2**	**3**

Prevalence is the proportion of cases in the studied sample. Relative Risk is the PPV/NPV ratio of the Target sample to the remainder. Target is the percent of the sample called positive. Effectiveness is the proportion of cases prevented by treatment (in the last two columns, assuming 75% in the target and 25% in the remainder). Percent of Preventable is the number of target cases prevented as a percentage of the number prevented if everyone were treated, given the indicated effectiveness. Percent of All Prevented is the number of cases prevented as a percentage of all cases. NNT All is the NNT for the total sample. NNT Targeted is the NNT in just the target sample. Abbreviations: NNT, number needed to treat; NPV, negative predictive value; PPV, positive predictive value.

Here, I consider these evaluations in the context of a review of five common conditions for which genetic risk assessment has been contemplated. An additional five conditions are discussed in S1 Text, which also includes further explanation of the measures of PRS performance, and S1 Table with relevant extracted estimates of drug efficacy. I then close with some recommendations for development and implementation of genetic risk assessment.

## Opioid use disorder

Over 80% of global consumption of prescription opioid pain killers (drugs that bind and act through one of the opioid receptors) is in the United States, which accounts for just under 5% of the world’s population [5]. It is estimated that almost 100 million US residents took an opioid last year, with between 5% and 10% of these people likely to become addicted or at least transition to opioid use disorder (OUD). This in turn helps explain the fact that upwards of 50,000 people died of a drug overdose in 2016, or 2% of all deaths in the US. An increasing proportion of this is now attributed to fentanyl, a synthetic drug initially developed for surgical sedation, which is 50 times more potent than heroin and is available off prescription (on the street) or laced into counterfeit prescription drugs. A microscopic increase in the dosage of fentanyl in one bad pill can be lethal to unsuspecting addicts just trying to cope with chronic pain [6].

There are three potential targets for prediction of opioid response. One is identification of individuals at high risk of addiction; another is identification of poor metabolizers, who have reduced conversion of ingested drug into a bioactive form like morphine and hence are not receiving analgesic benefits; and conversely, the third is identification of ultrarapid metabolizers, who produce so much active drug that they are at risk of serious adverse events, including respiratory depression, severe nausea and constipation, and dysphoria [7,8]. Guidelines for dosing are now provided by the Clinical Pharmacogenetic Implementation Consortium (CPIC) based on the known large effect of allelic variation at the *CYP2D6* locus on metabolism of at least codeine and tramadol and possibly oxycodone and hydrocodone [9]. Approximately 7% of the population fall into either the poor or ultrarapid metabolizer categories and may benefit from genetic assessment, but there are over 70 known SNP variants and copy-number variations, so genotyping is currently only performed at fewer than 10 medical centers in the US.

Prediction of OUD or addiction is a much more difficult problem [10]. Variants in the *OPRM1* gene, which encodes the μ opioid receptor expressed in the central nervous system, have been repeatedly associated with measures of response to pain medication, but these findings were from small studies and did not reach anything close to genome-wide significance [11]. Nevertheless, the San Diego company Proove Biosciences developed a test based on 11 SNPs broadly associated with addiction, mostly in neurotransmitter receptors and transporters, but the company was forced into receivership in August of 2017 after facing charges of kickbacks and over legitimate concerns surrounding the validity of the aggressively marketed test [12]. Extrapolation of their sensitivity analysis [13] on the assumption of 8% prevalence of OUD implies a precision of just 30%: in other words, the majority of the small percentage of patients recommended not to take opioids would actually not be at high risk of addiction.

By contrast, if a negative predictor could identify two-thirds of the population with half the risk of progression, allowing the social services and other health workers to give more resources and attention to monitoring of the one-third of higher-risk individuals, then successful intervention in just half of these cases would reduce the OUD rate by 25%. Alternatively, if the aim is to generate a test with a precision of at least 50%, then targeting 5% of the population would require a score defining this group with an odds ratio of 8.7, and highly effective treatment would also reduce OUD by 25% (see Table 2). It is remarkable that no genome-wide association study (GWAS) for opioid addiction based on hospital records has yet been reported and conceivable that phenome-wide association studies (PheWASs) [14] on a million patients will have the requisite power for development of a PRS.

**Table 2:** Approximate observed prevalence, relative risk, response, and the NNT.

Attribute	Opioid Use			CAD Events				Bone Fracture			
Prevalence	5	8	8	2.2/year	12	12	12	16	16	16	16
**Relative Risk**	**6**	**8.7**	**2**	**3.3**	**2.2**	**1.6**	**1.2**	**3**	**5**	**2.3**	**1.3**
**Target**	**1%**	**5%**	**33%**	**5%**	**20%**	**50%**	**80%**	**8%**	**10%**	**50%**	**80%**
**PPV/Precision**	**29**	**50**	**12**	**7**	**21**	**15**	**12**	**41**	**57**	**22**	**17**
**NPV**	**95**	**94**	**94**	**98**	**90**	**91**	**90**	**86**	**89**	**90**	**87**
**Accuracy**	**95**	**92**	**67**	**93**	**77**	**53**	**28**	**83**	**85**	**56**	**31**
**Sensitivity**	**6**	**31**	**50**	**15**	**35**	**62**	**83**	**21**	**36**	**70**	**84**
**Specificity**	**99**	**97**	**68**	**95**	**82**	**52**	**20**	**94**	**95**	**54**	**21**
**Effectiveness**	**80%**	**80%**	**50%**	**25%**	**25%**	**25%**	**25%**	**60%**	**60%**	**60%**	**60%**
**Percent of Preventable**	**6%**	**31%**	**50%**	**15%**	**35%**	**62%**	**83%**	**21%**	**31%**	**70%**	**84%**
**Percent of All prevented**	**5%**	**25%**	**25%**	**4%**	**9%**	**15%**	**21%**	**12%**	**21%**	**42%**	**50%**
**NNT All**	**25**	**16**	**25**	**182**	**33**	**33**	**33**	**10**	**10**	**10**	**10**
**NNT Targeted**	**4**	**2**	**17**	**61**	**19**	**27**	**32**	**4**	**3**	**7**	**10**

See Table 1 legend for explanation of terms. Data calculated from observed prevalence and projected relative risks from expected performance of genetic risk scores extrapolated from current data discussed in text. Abbreviations: CAD, coronary artery disease; NNT, number needed to treat; NPV, negative predictive value; PPV, positive predictive value.

## Hypertension

One billion people worldwide, including 75 million US residents, suffer from hypertension. It is well established that above 115 mmHg of systolic blood pressure (SBP), every increase of 20 mmHg doubles the risk of cardiovascular disease (CVD). There is much debate over guidelines, but the 8th Joint National Committee (JNC) report [15] called for a combination of lifestyle changes and pharmaceutical intervention—generally including a thiazide diuretic and possibly one or more of an angiotensin converting enzyme (ACE) inhibitor, or a blocker of the angiotensin receptor, beta-adrenoreceptor, or calcium channels—to keep SBP below 150, particularly in elderly persons over the age of 60. Formerly [16], this target was 140, or even 130 in patients with chronic kidney disease or diabetes, as there is strong evidence that doing so almost halves the risk of cardiovascular death. As of December 2017, the American Heart Association (AHA) now regards almost half of all US residents as in need of reducing their blood pressure [17,18].

A randomized control trial involving over 9,000 patients [19] evaluated the effectiveness of intensive therapy with an average of almost three drugs targeting SBP less than 120 mmHg compared with standard treatment with an average of two drugs targeting SBP less than 140 in elderly individuals with incident or preclinical heart disease (generally overweight and with a high Framingham risk score). It was stopped after just 4 years because of a clear benefit of intensive therapy showing a 25% reduction in primary end points (myocardial infarction, stroke, heart failure) from 2.19% to 1.65% events per year. Between one-quarter and one-third of these events led to cardiovascular deaths, which were also significantly reduced. Follow-up analyses [20] also revealed that there was no difference between the treatment arms in self-perception of effectiveness of medication (which can either be taken to mean that there is not likely to be any obstacle to adherence to intensive treatment or that there is no clear benefit to it in terms of general daily health). Furthermore, economic microsimulation showed that the average additional cost of intensive therapy of approximately US$1,000 per year, or around just 5% of the annual total cost of care for these individuals, is well below the “acceptable” threshold of US$50,000 per quality additional life-year (QALY) gained [21], given that median life expectancy is 12 years and an average extension of 3 months was estimated from the data. Such data argue for general introduction of intensive blood pressure control in this high-risk CVD population.

Table 2 shows that targeting intensive therapy to the 5% identified at highest polygenic risk, who have an odds ratio of 3.3 according to Khera and colleagues [2], would prevent 4% of all events, which is 15% of those preventable if everyone is treated. Two-thirds of the events could be avoided if half the population is treated aggressively. However, it also needs to be recognized that the rate of drug-induced, serious adverse events—mostly declining kidney function, often leading to chronic failure, syncope (fainting), and hypotension—was considerably higher (1.4× to 2.5×, depending on criteria) in the intensive treatment group than the decline in cardiovascular events [19]. Furthermore, the NNT for primary events per year is just over 60 for all-cause deaths, and cardiovascular deaths is double that, at around 15 over a decade. This means that the 3 months of QALYs per patient is more likely to reflect 1 in 15 or more patients avoiding an event such that they meet their normal life expectancy rather than meeting an early death, with ambiguous benefit to the remainder.

Meta-analysis of the FINRISK, Framingham cohort, and UK Biobank studies [22,23] has identified PRSs based on either 46,000 or 1.7 million SNPs for which the top and bottom quintiles are differentiated by 15 years in the age of cumulative incidence of 10% acute coronary events, with that threshold never met in low-risk women. Assuming a constant response rate to therapy of 25% for all genetic risk thresholds, targeting the top quintile would prevent one-third of deaths in men, halving the NNT (Table 2).

## Type 2 diabetes

The goal for therapeutic intervention in diabetes is not so much about prevention of disease onset as about prevention of progression to serious morbidities and mortality due mainly to CVD but also retinopathy, limb amputation, and internal organ damage. The frontline treatment has long been metformin [24], which reduces blood glucose for the most part by inhibition of mitochondrial gluconeogenesis. Its prescription to the vast majority of type 2 diabetics is largely based on perceptions following a 1998 study [25] implying a 35% reduction in mortality in overweight patients, reinforced by the 2002 United Kingdom Diabetes Prevention Program conclusion that metformin reduced diabetes incidence 18% over a decade [26]. The drug is well tolerated by over 80% of patients and relatively inexpensive at around US$50 a year, so even if only a minority of patients benefit, there is little perceived harm in widespread usage. However, meta-analyses have failed to confirm that there is in fact long-term benefit, with one prominent study in 2012 [27] concluding that the drug is as likely to increase all-cause mortality by 31% as to reduce it by 25%. Part of the difficulty is that the early analyses excluded patients also receiving sulfonylureas, which multiple studies have associated with elevated mortality and cardiovascular event rate and are given as supplementary therapy to patients whose glucose levels are not controlled by metformin (such nonresponders may be more likely to have poor outcomes) [27]. Similarly, the ACCORD study [28] found that intensive control of glycemia and lipids for long-term diabetics can increase all-cause mortality, emphasizing the importance of new strategies to guide prescription.

In the past decade, a new generation of drugs have emerged that act more broadly to reduce the so-called ominous octet of diabetes pathologies [29]: hepatic glucose production, pancreatic glucagon secretion by α cells, insulin secretion by β cells, neurotransmitter dysfunction, cardiac endothelial damage, reduced glucose uptake in muscle, elevated lipolysis, and renal glucose reabsportion. Because hyperglycemia is not thought to be the primary cause of cardiovascular pathophysiology, which is responsible for 80% of diabetes mortality, therapeutic intervention increasingly focuses on CVD. Agonists of the GLP-1 receptor and SGLT2 inhibitors have been shown in large randomized clinical trials [30,31] to reduce stroke, myocardial infarction, and cardiovascular death by approximately 20% in diabetic patients with CVD, independent of Hb1Ac (and, hence, blood glucose) reduction. Drugs like liraglutide (Victoza) and exenatide (Byetta) are currently over 100 times the cost of metformin. Although the price will presumably reduce when they become generic in the next few years, it would seem desirable to identify that subset of the diabetic population with CVD who are most likely to benefit and to assess whether patients at highest risk of CVD are more responsive. One study found that a lipid PRS did not differentiate metformin-responsive low-density lipoprotein (LDL) reduction [32], but it would be more informative to know whether the type 2 diabetes or CVD PRS identifies patients who are more likely to respond to the new generation of GLP-1 receptor and SGLT2 inhibitor drugs. Economic modeling has already argued that the combination of metformin plus a DPP-4 inhibitor, another drug that reduces mortality around 20%, though expensive at approaching ₤20,000/QALY gained [33], is within the accepted guidelines for cost-effectiveness.

Given that adiposity, measured both generally as body mass index (BMI) and centrally as waist-to-hip ratio (WHR) adjusted for BMI (WHRadjBMI), is strongly associated with metabolic syndrome, it has been argued that weight reduction is among the most important public health goals for the coming decades. Mendelian randomization studies of the UK Biobank with a PRS genetic instrument explaining approximately 2% of BMI indicate that an increase of one standard deviation unit (approximately 5 kg/m^2^) causally doubles the risk of type 2 diabetes [34], and similar results are seen in the DIAGRAM study [35] for WHR, with an independent PRS explaining approximately 1% of the trait. Although it is notoriously difficult to maintain a 10% reduction in weight long term, recent studies indicate that intensive lifestyle intervention is effective and can produce remission of diabetes [36]. Moreover, even moderate exercise significantly reduces cardiovascular mortality across urban and rural settings in low-, moderate-, and high-income countries [37].

## Depression

Major depressive disorder (MDD) has rapidly become the second-leading source of morbidity globally. At least 1 in 20 people will lose meaningful quality of life-years to depression, and total costs to the healthcare system alone in the US have been estimated at up to US$100 billion per year [38]. Even though meta-analyses provide little indication that antidepression medications (ADMs) lead to better rates of long-term remission than behavioral therapies [39,40], there are several reasons why the adoption of ADMs is only likely to increase in the coming years: the short-term benefits for symptom relief are superior, there is a severe shortage of trained therapists, and insurance coverage for mental health is unreliable [41]. However, only around half of patients will respond to the first ADM they are prescribed, and most of the two-thirds who do benefit will need to test three to five drugs before finding a regimen that is both effective and tolerated. Including hospitalization, this may cost tens of thousands of dollars, so once again, there are enormous economic (and clinical) benefits to be gained from genomic predictive evaluation.

The heritability of MDD (at about 30%) is one of the lowest of all common disorders, in part reflecting broad heterogeneity in environmental contributions, and accordingly, GWAS has made few inroads. Despite a sample size including 120,000 cases, the largest studies to date have discovered just 15 replicated loci for MDD [42], and a genome-wide PRS explains less than 1% of the variance [43] and thus shows little predictive potential. An even larger GWAS for neuroticism [44] turned up 116 independent loci, and the polygenic score is highly correlated with that for depression, but it still explains just a small fraction of the variance. Furthermore, the genetic correlation between Asian- and European-ancestry populations [45] is notably less than 1, and somewhat differential genetic risk is implicated for early- and late-onset disease [46].

Similarly, GWAS has not yet identified any replicated genome-wide significant loci for ADM response [47,48], even though common variants explain over 40% of the variance. There is some preliminary evidence [49] that quartiles of polygenic scores for openness, neuroticism, and conscientiousness differentiate response to selective serotonin reuptake inhibitors (SSRIs) and possibly predict remission, but differences in sign of effect at 4 and 8 weeks of treatment caution that many more than 1,000 subjects need to be evaluated. There is also an extensive body of literature evaluating two dozen candidate genes for pharmacogenetic modulatory effects. These mostly target serotonin production and signaling (because they are the targets of the major classes of ADM) as well as drug metabolism and bioavailability. Meta-analyses [50,51] implicate variation in the serotonin transporter and receptors (the 5HTTLPR in *SLC6A4*, *HTR2A*) as well as cytochrome P450s and a few other loci, though with apparent heterogeneity across ethnicities, unexpected effects such as a heterozygote advantage for V66M in *BDNF*, and overall lack of Bonferroni-adjusted significance. It should be noted that, with odds ratios in the vicinity of 1.5, current sample sizes of fewer than 3,000 patients are underpowered, so much larger pharmacogenetics GWASs are urgently required. Despite this, CPIC has released guidelines for implementation of pharmacogenetics for SSRI dosing based on CYP2D6 and CYP2C19 genotypes [52], and at least four companies are offering combinatorial pharmacogenetics diagnostic tests (CPGx: the Neuropharmagen [53], GeneSight [54], Admera PGxPsych, and MD labs Rχight panels). I could not find publications describing clinical assessment of the latter two, but the first two both show meaningful improvements in 12-week response (for example, reducing depression symptoms in 50% of patients when physician-ordered ADMs are congruent with test predictions, relative to 35% of those who are not given a CPGx test and 39% for all tested patients regardless of congruence). One of the companies also reports cost-effectiveness of the test, saving almost US$4,000 in medication costs per patient treated solely by a primary care provider, relative to US$2,500 for the one-time diagnostic [55]. Because these are proprietary tests, it is impossible from the published data to know which variants are most useful, and clearly much larger independent studies will be needed to comprehensively evaluate utility across dozens of drugs. It is highly likely, though, that much of the benefit comes from the negative predictive value of avoiding ADMs that are predicted either to lead to intolerable side effects or not to alleviate symptoms. Potential healthcare savings in this domain run plausibly to tens of billions of dollars per year once validated mature pharmacogenetic tests are available.

## Osteoporosis

In the US alone, over 1.5 million postmenopausal women suffer clinically significant bone fractures each year. Lifetime risk of both hip and vertebral fractures is over 15% [56], with hip fracture seriously impeding the mobility of half of the patients for an extended period of time, leading to a requirement for long-term ambulatory care in a third of cases. It is not clear whether the 10% elevation in mortality after a fall leading to hip fracture is a consequence of fracture or comorbid with underlying disease. Hormone replacement therapy with estrogen and progesterone was consistently found to reduce fracture rates by around 40%, but following the Women’s Health Initiative’s [57] detection of meaningfully elevated rates of invasive breast cancer and cardiovascular morbidity, it is no longer widely adopted. Instead, bisphosphonate inhibitors of bone resorption such as alendronate [58], which are similarly effective without the serious adverse effects, have become standard of care, also showing cost-effectiveness in high-risk groups over the age of 70 [59].

Recently, a monoclonal antibody inhibitor of sclerostin that simultaneously increases bone formation and decreases bone resorption, romosozumab, was shown in two randomized clinical trials to reduce both vertebral and nonvertebral fracture rates [60,61]. Direct comparison of this treatment with alendronate alone in high-risk women with bone mineral density (BMD) in the bottom decile and a history of fracture demonstrated remarkable efficacy consistent with an NNT for vertebral fracture of less than 20 (6.2% relative to 11.9% over a 2-year follow-up, which would likely be around 10 relative to placebo). However, the US Food and Drug Administration (FDA) has disallowed registration of the drug because the data show a worrying elevation of the serious adverse cardiovascular event rate from 1.9% to 2.5%. A Japanese study argued that as few as four SNPs can differentiate high- and low-risk deciles for vertebral fracture [62] by as much as 10-fold. Concerted robust genomic and clinical evaluation thus has potential to stratify patients for treatment: if a polygenic score achieves a relative risk of five for the top decile, then 20% of cases could be prevented with a precision of over 50%. An analysis of the UK Biobank for osteoporosis and general bone fracture is less promising [63] but did not target the low-BMD group. In any case, genetics could rescue a treatment that has the potential to prevent loss of quality of life for hundreds of thousands of women each year while also ensuring that the costs to the healthcare system are contained.

## Discussion

With the advent of clinically relevant polygenic scores [2], the time has come for research designed not just to identify high-risk groups but also to evaluate therapeutic response rates across the distribution of scores. The oft-reported measures of sensitivity and specificity are not generally likely to be clinically useful, as scores that identify the upper two deciles of the risk distribution generally have sensitivities less than 50%. Fig 1 assumes case-control comparisons but might actually be more informative in relation to the prediction of therapeutic responses in patients already diagnosed with disease. In some situations, response may be highly correlated with risk of disease, but that cannot be assumed, so more GWASs of disease progression therapeutic response are needed.

Although it is often assumed that all patients should receive treatment, considerations of cost, adverse side effects, development of resistance, and patient choice all mitigate in some cases toward preferential treatment of the patients at highest risk of progression to life-threatening disease and/or most likely to respond to therapy. Meaningful reductions in the NNT while ensuring high prevention rates will be most attainable if therapy can be targeted to individuals predicted to have the highest response rates. It should generally be possible to prevent 20% to 50% of cases by treating between 5% and 20% of the patients. Conversely, negative prediction may identify those least likely to benefit, which is likely to become increasingly important in relation to expensive next-generation biologics and in markets emphasizing cost control.

Two further critical components of this evidence-based approach to clinical intervention are the costs of treatment and patient rights. Economic modeling of healthcare costs is notoriously difficult, but it is nevertheless clear that either the government or private health insurers must bear the short-term costs of expensive new-generation medications such as biologics. Any consequent rationing of options must be evaluated alongside patient rights and desires. Box 1 argues that some individuals are likely to demand treatment irrespective of where they fit in terms of risk if the drug has been approved for general use; others may prefer not to take drugs as far as possible and will see genomic information as empowering [64, 65]. Genetic risk scores are an important step toward personalized genomic medicine and will need to be implemented with respect to the specific clinical and economic circumstances of each disease.

**Box 1. Genetic risk assessment for common disease**

This Review makes three major claims: (1) that evaluation of the appropriate level of prescription medication usage should be a public health priority, (2) that implementation of this policy will vary widely according to disease and therapy, and (3) that genomic diagnosis has an important role to play in stratifying need for specific drugs. There are two broad domains that should be considered: prevention and control of common chronic diseases and treatment of acute, less-common diseases with expensive new-generation drugs and biologics. The latter engage economic issues that are largely the concern of medical providers; it is the former that presents the most prospects for patient-driven solutions.

A typical situation involves a middle-aged adult who learns that he/she has elevated biomarkers indicative of high risk for heart disease. Because medical guidelines call for reduction of cholesterol or blood pressure in such cases, a medication is prescribed and almost always accepted. Over the next several months, dosages and specific medications are adjusted to alleviate negative side effects such as nausea and poor mood, or the patient adjusts to the discomfort. Within a couple of years, many will have become noncompliant, with unknown consequences for future care. One response is to develop technologies that track and enforce compliance; another is to engage in thoughtful discussion of whether prescription medication is most appropriate.

To this end, we might envisage all prescriptions requiring three levels of conversation with the patient. The first is a comprehensive discussion of adverse outcomes, not just of labeled serious risks and drug interactions but also of prevalent negative impacts on quality of life. There is likely to be wide variation in the extent of such doctor–patient conversations currently. The second is understanding of the concepts of risk stratification and of precision, including acceptance that if a prescribed drug prevents 20% of primary outcomes, then 80% of such events still occur. Either a large proportion of patients do not respond to the treatment or the response (lower blood pressure, cholesterol) is not sufficient to prevent the cardiovascular event, and there is patient-to-patient variability. The third is to aim to empower patients to make their own decisions about whether their total medical profile places them in a risk category for which the benefit of medication is sufficient to overcome side effects, expense, and inconvenience. Some people will choose to do all they can to prevent an event (no one wants to be denied or refuse treatment only to develop a life-threatening state), whereas some will prefer not to go on medications if they can possibly avoid them (recognizing that no treatment is anywhere near guaranteed to prevent progression). Clearly, extensive physician and patient education and communication will be required to optimize patient engagement.

Although it is likely that the majority of people will continue to choose medication, the availability of validated risk stratification could have an important influence on future prescription rates. A major goal for genomic medicine should thus be the development of predictive scores across the full spectrum of disease, integrating genetic evaluations of risk of disease, risk of disease progression, and response to therapy alongside best-practice clinical profiling.

## Supporting information

S1 Table NNT for select published therapeutic responses. NNT, number needed to treat.
(DOCX)

S1 Text Further background and five additional conditions. (DOCX)
