# Supplementary material for: On the utilization of polygenic risk scores for therapeutic targeting
Source: PLoS Genet. 2019 Apr 25;15(4):e1008060. doi: 10.1371/journal.pgen.1008060 (PMC6483161; doi:10.1371/journal.pgen.1008060)
Supplement: S1 Table — NNT, number needed to treat. (DOCX) [file pgen.1008060.s001.docx]

**S1 Table NNT for Select Published Therapeutic Responses**

**Comparison Outcome N Control Treat RRR ARR NNT Study Ref**

Standard vs Intensive

BP Treatment MI, Stroke, HF or CV-death / yr 9361 2.19% 1.65% 25% 0.54% 185 SPRINT (2015) Table 2 21

Standard vs Intensive

BP Treatment MI, Stroke, HF or CV-death / 3¼ yrs 9361 6.81% 5.19% 24% 1.62% 62 SPRINT (2015) Table 2 21

Standard vs Intensive

BP Treatment CV Death / 3¼yrs 9361 1.39% 0.79% 43% 0.60% 167 SPRINT (2015) Table 2 21

Standard vs Intensive

BP Treatment Serious Adverse Events / 3¼ yrs 9361 11.8% 15.9% 35% 4.1% 24* SPRINT (2015) Table 3 21

Control vs Statin Major Vascular Event / yr 175000 4.04% 3.27% 24% 0.77% 130 CCT Meta (2015) Fig 1 25

Control vs Statin

(<5% 5yr MVE risk) Major Vascular Event / yr 24790 0.56% 0.38% 32% 0.18% 555 CCT Meta (2015) Fig 1 25

Control vs Statin

(>30% 5yr MVE risk) Major Vascular Event / yr 21679 9.82% 7.64% 22% 2.18% 46 CCT Meta (2015) Fig 1 25

Control vs Statin Vascular Death / yr 175000 1.30% 1.47% 13% 0.17% 590 CCT Meta (2015) Fig 3 25

Control vs Statin (top 20% GRS) First CHD Event, 13yr follow-up 988 19.6% 11.7% 44% 7.9% 13 Natarajan (2017) Table 3 30

Control vs Statin (0-80% of GRS) First CHD Event, 13yr follow-up 3910 12.9% 10.1% 24% 2.8% 36 Natarajan (2017) Table 3 30

Control vs Metformin in T2D All cause-death (1yr follow-up) 8732 1.3% 1.1% 16% 0.2% 500 Boussageon (2012) Fig. 2¶ 35

Low vs High physical activity All-cause-death/1000 person yrs 81274 6.4% 4.1% 36% 2.3% 43 PURE (2017) Table 3 44

Low vs High physical activity CV-death / 1000 person yrs 81274 1.8% 0.9% 50% 0.9% 111 PURE (2017) Table 3 44

Low vs High physical activity Death or CVD / 1000 person yrs 81274 9.5% 6.6% 30% 2.9% 34 PURE (2017) Table 3 44

CPGx test Major Depression Sustained response to medication 316 34.4% 38.5% 12% 2.9% 24 Pérez et al (2017) Figure 2 64

Congruent PGx test for MDD 12 week response to medication 299 51.3% 36.1% 42% 15.2% 7 Pérez et al (2017) Figure 3 64

Alendronate vs romosozumab Bone fracture in low BMD ♀ / 24mo 3150 11.9% 6.2% 48% 5.7% 18 ARCH (2017) Figure 2 80

Alendronate vs romosozumab Serious cardiovascular event / 12mo 4054 1.9% 2.5% 31% 0.6% 167* ARCH (2017) Table 2 80

Non-biologic vs anti-TNFα in CD Progression to penetrating disease 382 5.8% 2.1% 64% 3.7% 27 RISK (2017) Table 2 89

Abbreviations: RRR, Relative Risk Reduction; ARR, Absolute Risk Reduction; NNT, Number needed to treat

* These two entries represent the NNH (Number needed to harm) ¶Specifically, entry for COSMIC study in Cryer D, et al (2005) Diabetes Care 28: 539-543.
