# Supplementary material for: On the utilization of polygenic risk scores for therapeutic targeting
Source: PLoS Genet. 2019 Apr 25;15(4):e1008060. doi: 10.1371/journal.pgen.1008060 (PMC6483161; doi:10.1371/journal.pgen.1008060)
Supplement: S1 Text — (DOCX) [file pgen.1008060.s002.docx]

**S1 Text: Further Background and Five Additional Conditions**

***Sensitivity, Precision, and the Number Needed to Treat***

Personalized medicine promises to target the right medication to the right person at the right time [66]. Whereas generic drugs may only alleviate pathology for say a half of all patients, according to the positive narrative which accompanies genomic medicine, personalized treatment aims to target effective response for the majority [67,68]. Yet polygenic scores only identify a minority of individuals at high risk, which begs the question of how they should be best utilized to maximize benefit.

It is common for genetic studies to report the “area under the receiver operating curve” (AUC) as the primary measure of performance of a new test such as a polygenic risk score (PRS). This probably derives from the desire to develop a score that performs well in discriminating cases from controls, namely one that is diagnostic of most people who have a condition while not calling too many false positives. Sensitivity is the fraction of called positives who truly have a condition, while specificity is the fraction of called negatives who do not have it. It is always possible to develop a test with high sensitivity, but which is not useful if the specificity is low. By analogy, hair length greater than 3cm may correctly identify 90% of all women, but also 50% of all men (hence have high sensitivity and low specificity); whereas a hair length cutoff of 20cm may have a specificity of 90% since few men have hair that long, but sensitivity of just 50% since many women have shorter hair. The AUC essentially averages over all possible cutoffs and if greater than 80% is often of clinical utility, where 50% is no better than random guessing. Ultimately, though, a specific cutoff must be chosen as the basis for a diagnostic test, based on the optimal balance of sensitivity and specificity that produces high “accuracy” (the total proportion of true positives and true negatives in the sample).

Getting that balance right reads well in a journal, but from the patient’s perspective is often not relevant. What a patient and their physician should want to know is usually the precision, also known as the positive predictive value. This is the proportion of people called positive for the score, who actually are. Precision depends on the incidence of the condition, and rarity reduces the value. It is for example possible to have extremely high sensitivity and specificity, both over 99% as is the case for assessment of trisomy from maternal blood DNA sequencing [69], yet precision of just 50% if the condition is very rare, say 1 in 10,000. Alternatively, modest accuracy can nevertheless have quite high precision if the condition is common, such as meaningful lowering of cholesterol on taking statins. Since most diseases are rare, most genotype-based tests have low precision; and similarly clinically meaningful drug responses may also be rare. The objective then is to use such tests under circumstances where the ratio of cases to controls is elevated, for example in affected families, or after excluding as many non-cases a priori as possible. This is similar to increasing the negative predictive value, namely using a threshold that correctly identifies that portion of the population who definitely will not be cases.

The Number Needed to Treat (NNT)[4] is a different concept. Rather than focusing on the relative risk reduction (RRR), it uses the absolute risk reduction (ARR) to infer how many patients need to be treated in order to avoid one adverse event. Consider two situations where the RRR is 20%, namely from 2% to 1.6%, or from 20% to 16%, then the ARRs are 0.4% and 4% respectively. The NNTs are the inverse of these percentages, namely 250 and 25. For very rare conditions, even though a treatment results in a significant reduction in incidence, it takes hundreds of people to take the drug in order to see one benefit, and even for quite common conditions the NNT can be above 20. One interpretation is that most people taking the drug are not actually benefiting, that the 20% reduction in mortality is more because one person lives 10 years longer than they otherwise would have, rather than most patients living 3 months longer. A drawback of the NNT is that it is sensitive to the time window: the above numbers might also correspond to one year and ten year survival rates on a drug, where the number of people benefiting becomes much greater over time. Unfortunately, there are few studies monitoring the very long term benefits of even some of the more common prescription drugs, and given low compliance rates as well as confounding of all-cause with disease specific mortality in aging cohorts, these are difficult to evaluate.

Most commonly prescribed heart and cholesterol medications listed on the [www.thennt.com](http://www.thennt.com) website have NNT over 100, and many have corresponding numbers needed to harm (NNH) in the 10s, implying that many more people are harmed than benefit with respect to hard outcomes like death and coronary event. The NNT perspective thus also calls for evaluation of the most appropriate target populations for medication, rather than blanket prescription for all candidates. In the main article I discussed how genomics might be used to balance the desires for high precison and low NNT for five representative diseases. Here I discuss five additional diseases, not all of which currently have polygenic risk scores available, yet collectively illustrate some other considerations in the use of genomic prediction.

**Five Additional Conditions where Genomics might Guide Therapeutic Intervention**

*Hyperlipidemia*

Statins (anti-HMG coA reductase inhibitors) are extraordinarily widely used by as much as 15% of the adult US population to reduce the levels of low density lipoprotein (LDL) in the blood. Long-term guidelines recommended by the ATP III report in 2001 [70] focused on specific targets for reduction of LDL, noting that the association of LDL with cardiovascular events is linear in the range from baseline levels of 115 up to 200 mg/dL and that statins are generally effective in reducing LDL levels by 40 mg/dL (~1 mMol/L) or more. Since meta-analysis of 27 randomized control trials with 175,000 participants [71] has shown that each increment of 1 mMol/L in circulating LDL is associated with a 21% relative increase in major cardiovascular events, which are responsible for one fifth of US mortality, widespread statin usage is regarded as a high public health priority. More recent guidelines [72] instead call for classifying people in high, intermediate and low risk groups, and taking into account other patient-specific factors, but there is ongoing debate, with some authors observing that since CVD events, including deaths, are decreased to a similar degree across all baseline LDL levels, perhaps all people should consider statins. Counterbalancing this, a Dutch modeling study [73] found that despite the low costs of generic statins, the total cost of their use including doctor visits, lab tests and other fees is such that treatment for low-risk groups is not cost-effective, rising above the $50,000 per QALY threshold.

In contrast to anti-hypertension medication, the serious adverse event rate (primarily life-threatening rhabdomyelysis and myotrophy leading to kidney disease) is significantly lower than the observed rate of protection from primary CVD events. Nevertheless, statin usage itself is subject to high rates of non-adherence, with over 50% of individuals (possibly up to 75%) no longer taking the drug 2 years after initial prescription, including in patients recovering from a major event [74]. This may have to do with costs, administrative issues, and lack of understanding or perception of benefits, but is likely primarily due to the fact that there are many negative side-effects such as pain, fatigue, diarrhea, and dizziness, all subject to warnings in advertisements for Lipitor, Crestor, Zocor and the like. Basically, quality of life is reduced, even if this does not reach the level of a medical event. If the perception is of no benefit, reflecting the low probability of CVD events to begin with, then it again makes sense to target therapy to those who are most likely to benefit.

To this end, two genetic risk score studies have clearly demonstrated enormous potential. Both a 27 SNP [75] and 57 SNP [76] predictor based on independent associations with cardiovascular disease or MI show very clear discrimination of CVD events in multiple randomized clinical trials. Individuals in the top quintile of genetic risk are 70% more likely to have a major CVD event than those in the bottom quintile, and a 25% increase in incident heart disease is observed for each standard deviation increase in PRS. Absolute risk reduction due to statin therapy is significantly improved in the high risk quintile for whom the NNT is 28, compared with 80 for the remainder. This is due both to a higher baseline risk as well as a better response rate likely because those SNPs predict coronary calcification and plaque development independent of lipid levels. It is not yet known whether the combination of low genetic risk and low LDL has particularly low statin response.

*Attention Deficit-Hyperactivity Disorder*

Alongside cardio-metabolic disease, pharmacological intervention is most prescribed for neuro-psychological ailments. Since the 1990s, one of the most medicated conditions has been aberrant attentional, language usage, and activity issues that are now collectively referred to as Attention Deficit-Hyperactivity Disorder, ADHD. Current estimates are that at least 5% of children could be diagnosed with ADHD according to DSM-5 criteria [77], and that up to half of these cases are medicated in the US, compared with one tenth in Europe. Frontline therapy is the psychostimulant methylphenidate (MPH, best known as Ritalin) which inhibits the reuptake of dopamine in synapses. Amphetamines and non-stimulant drugs are also commonly used, all usually in conjunction with behavioral interventions. Studies of efficacy are confounded by heterogeneity of diagnosis, of study populations and of medication regimens; by small sample sizes; and by a focus on short-term responses. Nevertheless, it is apparent that over 70% of ADHD subjects remain on MPH for a year or more, and that the drug leads to significant reductions in objective measures of ADHD-related symptoms in at least 40% of cases [78]. At least 15% of subjects go off medication, usually within several weeks of initiation, due to inability to tolerate side-effects such as loss of appetite (or anorexia), insomnia, anxiety and in some cases cardiac problems. Serious adverse events are thought to be rare, and overall the impression from the clinical literature is that ADHD medications are generally safe and effective, and sufficiently inexpensive ($50 per year for the fast-acting thrice-daily pills) that widespread usage is both cost-effective and beneficial.

However, there are unresolved concerns. A major one is that there is very little literature evaluating long-term consequences of Ritalin or other therapies. One recent observational study of adults with ADHD found ongoing benefits of MPH at least a year after administration of a slow-release once-a-day formulation [79] which seems to have better compliance, and to be more efficacious [80]. But given that other drugs targeting the dopamine transporter, notably cocaine, rewire neurotransmitter gene expression after long-term usage, leading both to dependency and resistance, there is legitimate concern [81] that five or more years of MPH may be detrimental and/or interfere with natural processes of resolution of symptoms which occurs in over half the cases. Secondly, the primary endpoint of most efficacy studies is reduction of symptoms on one or more validated questionnaire scales. The number needed to treat by such measures is less than 3, much as is reduction of blood pressure by anti-HT drugs is, but this is not necessarily as appropriate a measure as a quality of life outcome, comparable to heart attack or stroke, would be. Rates of hospitalization due to self-injury, graduation from College, maintenance of stable partnerships (or conversely, unusual levels of achievement) have not been objectively measured in randomized studies that I was able to find. Further, children seem to see less value in medication than their parents do [80], so it would be forgivable to conclude that the major beneficiaries of widespread MPH use are parents and teachers. In fact, loss of income or employment for primary caregivers, usually mothers, may be a much greater expense than medication per se [82], illustrating how difficult it is to evaluate the medical and cost-effectiveness of psychostimulation for ADHD.

Against this background, it is also difficult to see how genomic analysis might be used to target medication to those most in need. Despite high heritability above 70% estimated from twin studies [83], individual genotype effect sizes are small. GWAS including 20,000 ADHD individuals identified just a dozen loci [84], and a polygenic risk score including 50,000 SNPs explains less than 1% of the variance in two large population studies for both case-control status [85] and continuous attentional or activity measures [86], so does not yet have predictive utility. Underscoring the complexity of this condition which is perhaps best regarded as the extreme of a continuous distribution of symptoms than a discrete trait, is the significant genetic correlation with numerous measures of educational attainment, personality, body weight, lipidemia, smoking, insomnia and age at menopause [84].

*Epilepsy*

Epilepsy is another disorder where pharmacogenetics has long been envisioned as an important component of therapeutic decision making [87]. However, the reality has not met initial expectations, likely due to a combination of factors: considerable heterogeneity of disease presentation (focal, generalized, encephalopathic, and non-convulsive epilepsies all have different mechanisms), lack of replicated candidate gene findings from large RCTs (with the exception of a splice-site variant in *SCN1A* that modulates sodium-channel blocker effectiveness [88]), and clinician perception that genetic associations are not clinically useful. Approximately 70% of patients respond to at least one of dozens of anti-epileptic drugs (AEDs), which typically act quickly and can be dosed to bring patients into the therapeutic range readily. Although a quarter of patients are likely to go off therapy due to side-effects, there does not seem to be a need to predict these genetically, but for a couple of exceptions [89]. One is the HLA-B*15:02 allele which is relatively common only in south-east Asia but almost diagnostic of a potentially life-threatening inflammatory reaction to carbamazepine known as Stevens-Johnson Syndrome, and another is *POLG1* mutations which associate with hepatic failure in patients treated with valproate. Screening of the former would save the Taiwanese medical system close $1B a year if all patients were denied the less expensive drug out of concern over serious toxicity [87].

Recent research in this field has switched to integrative genomics and cell biology with a focus on pharmacodynamics, namely by understanding and intervening with the actual molecular lesion in each patient. Epilepsy has high heritability and at least 5% of cases can now be attributed to a copy number variant, or a *de novo* or inherited rare variant detected by trio exome or genome sequencing. Mutations in almost 75 genes were reported to contribute to pathology in a highly penetrant manner in a 2015 review [90], and these fall into classes such a sodium channels, nicotinic acetylcholine receptors, mTOR deficiencies, and cell polarity defects, each of which suggests a different treatment modality. However, there is unlikely to be a one-to-one gene-to-drug relationship since different mutations have different effects, and the genetic and environmental background modulate gene activity [91]. A combination of single-cell, neuronal culture, and animal modeling of each case has been proposed as a personalized medicine framework [86]. Whether this will support therapeutic decision making for the high percentage of less severe cases which are less likely to be resolved by genome sequencing remains to be seen.

However, effective interventions such as placing patients with disrupted SLC2A1 function on a ketogenic diet [92], or with aberrant ALDH7A1 activity on vitamin B6 (pyridoxine)[93], point toward a precision medicine future that is indeed deeply personalized. It does seem likely that even where a pathogenic mutation cannot be pinpointed, modeling of each case will often highlight the pathway that is affected. This may not always lead to efficacious prophylaxis, but it will generally exclude large classes of candidate drugs from consideration, once again showing the potential benefit of a negative predictive genetic evaluation.

*Plaque Psoriasis*

I turn now to discussion of two examples of targeted therapeutics based on the use of monoclonal antibodies (mAbs) to reduce the activity of key proteins. A third example discussed in the main text arose from the discovery of rare loss-of-function variant in the SOST gene (encoding the Sclerostin protein) that is protective against osteoporotic bone fracture [94]. Of course, precision cancer therapy increasingly often utilizes biologics, including immunotherapies targeting checkpoint inhibitors such as CTLA-4, PD-1 or its ligand, PDL-1 [95]. These biologic therapies are two to three orders of magnitude more expensive than generics, generally costing in the range of one to three thousand dollars per biweekly or monthly treatment hence rising to over $50,000 per year for medication alone. They are thus presumably not suitable for widespread adoption, and genomics is expected to pave the way to identification of subsets of patients who are most likely to respond.

Plaque psoriasis is an inflammatory autoimmune skin condition that negatively impacts quality of life for up to 4% of the adult population. Mild cases are generally treated with topical ointments and vitamin or photo-therapy, whereas moderate to severe cases have traditionally been treated with the immunosuppressant medications methotrexate and cyclosporine. These are not tolerated by 30% of patients due to nausea and hepatotoxicity and are not necessarily effective in the long term [96]. As it became clear that psoriasis is not just a disease of keratinocytes, but has a strong auto-immune component, initial biologic therapy targeted the Th1 cytokine TNFα by way of the synthetic decoy receptor etanercept (Enbrel) or inhibitory monoclonal antibodies infliximab (commonly Remicade) and adalimumab (Humira)[97]. Subsequent mAb strategies target the Th17 arm of the regulatory T cell response, initially ustekinumab (Stelara) against the p40 subunit of the IL-23 receptor that is shared with IL-12, and most recently risankizumab (BI 655066/ABBV 066) against the p19 subunit of IL-23R [98], or brodalumab (Siliq/Kyntheum) against the IL-17 receptor [96], and ixekizumab (Taltz) and secukinumab (Cosentyx) against IL-17 itself. Others are also in development, but it is notable that several of these mAb have been reported in phase III trials to achieve at least 80% success, defined as 75% reduction of the Psoriasis Area and Severity Index, PASI-75 within 12 weeks of administration. Concerns over suicide ideation in fewer than 1% of brodalumab recipients, including a handful of completed suicides, have been addressed with a “black box” warning that accompanies prescription. In all cases, well over half of all patients experience adverse events that may or may not be drug-related, only a small minority of which are considered serious enough to be life-threatening [99]. Upper respiratory and fungal infections and bowel disease are common side-effects, and it is not yet known what the consequences of long-term usage, including remission rates, are.

Current treatment is based on a combination of patient and physician preferences, severity and location of lesions, and comorbidities, rather than genetic or other biological signatures. However, given the expense of biologics there is a pressing need for development of predictors of likely curative and adverse responses. Targeted genotyping of a handful of candidate genes in small pilot studies suggests that significant associations will be identified that may be useful [100], but no approved pharmacogenetic platform for autoimmune therapy is yet available. Gene expression profiling has also been considered, since microarray analyses have shown that many of the therapies mentioned above lead to reversion of affected skin profiles toward normality, but with variable efficiency: for example, ixekizumab seems to be much more effective than etanercept in alleviating molecular signals of inflammation [101]. However, no prospective analysis of whether baseline gene expression predicts response to specific drugs has been reported. It seems unlikely that a positive predictor favoring one mAb over another will emerge, but prospects for negative prediction of adverse outcomes including failure to respond are quite plausible. In this regard it is relevant to note the results of longitudinal whole peripheral blood gene expression profiling of a lupus cohort [102] where corticosteroids and mycophenolate mofetil (MMF) elicited neutrophilic and plasmablast responses to varying degrees among patients. Eight classes of transcriptional response reflecting different modes by which the immune system correlates with disease severity were observed, but more studies are required before it can be concluded that these predict therapeutic outcomes.

*Inflammatory Bowel Disease*

Crohn’s Disease and ulcerative colitis jointly affect over 1% of adults in industrialized countries, including an estimated 3 million Americans [103]. Two major environmental risk factors have been implicated, namely cigarette smoking and changing patterns of intestinal microbiota, possibly linked to antibiotic usage [104]. Despite a typical course of remission with flares, 80% of patients are expected to require surgical resection within 20 years of diagnosis, and 30% within 5 years – particularly children in whom disease progression is thought to be more rapid. Consequently, the therapeutic goal of medication usage is attainment and maintenance of steroid-free remission in the hopes of preventing the need for colectomy. The anti-inflammatory drugs mesalamine (5-ASA) and either azathioprine (AZA) or 6-mercaptopurine (6-MP) are somewhat effective and are commonly used for maintenance therapy, but have side effects, variable compliance, and do not prevent advance to complicated disease for a large proportion of patients [104]. Corticosteroids by contrast are used to induce remission, but can increase rates of infection as well as metabolic comorbidity, and are only consistently effective in moderate disease.

Two forms of disease complication, stricturing fibrosis, and penetrating or fistulating extreme inflammation are observed. A recent prospective cohort study [105] utilized propensity score matching to conclude that anti-TNFα therapy when introduced soon after diagnosis is effective at preventing progression to penetrating disease, but probably not fibrosis. Again, given the high cost of the mAbs, their use as first line therapy is questionable and screening to identify the subset of patients most likely to respond would be highly desirable. In this regard, we noted that ileal gene expression at diagnosis is meaningfully predictive of progression to complicated disease (it is said to be prodromal, since aberrant gene activity already manifests prior to visible progression), and that the combination of gene expression signature with a serological score identified one third of the cohort who remained in remission [105]. If replicated independently, this approach could effectively target mAb therapy only to those most likely to require it. Furthermore, the suggestion of higher TNF-pathway gene activity might also identify patients at highest risk for penetrating disease. In parallel, high pre-treatment expression of Oncostatin M has been shown to presage emergence of anti-TNFα resistance [106]. Since genetic risk scores for inflammatory bowel disease, summing over more than 200 genome-wide significant loci, are poor predictors of disease progression [107], these studies highlight the potential of integrative genomic approaches. It will be interesting to see whether they are also useful in relation to selective adhesion molecule inhibitors such as vedolizumab (Entyvio)[108] which reduce inflammation by blocking migration of T cells to the gut.

**Additional References**

66. Torkamani A, Andersen KG, Steinhubl SR, Topol EJ. High-definition medicine. Cell 2017; 170(5): 828-43. PMID: [28841416](https://www.ncbi.nlm.nih.gov/pubmed/?term=28841416).

67. Auffray C, Charron D, Hood L. Predictive, preventive, personalized and participatory medicine: back to the future. Genome Med. 2010; 2(8): 57. PMID: [20804580](https://www.ncbi.nlm.nih.gov/pubmed/?term=20804580).

68. Ginsburg GS, Willard HF. Genomic and personalized medicine: foundations and applications. Translational Med. 2009; 154(6): 277-87. PMID: [19931193](file:///C:\Users\ggibson3\Documents\Manuscripts\Negative%20NRG\PLGE\19931193).

69. Yang H, Xu HB, Liu TT, He XL. Systematic review of noninvasive prenatal diagnosis for abnormal chromosome genetic diseases using free fetal DNA in maternal plasma. Genet Mol Res. 2015; 14(3): 10603-8. PMID: [26400291](file:///C:\Users\ggibson3\Documents\Manuscripts\Negative%20NRG\PLGE\26400291).

70. National Cholesterol Education Panel. Third Report of the National Cholesterol Education Program (NCEP) Expert Panel on Detection, Evaluation, and Treatment of High Blood Cholesterol in Adults (Adult Treatment Panel III) final report. Circulation 2002; 106(25): 3143–421. PMID: [12485966](file:///C:\Users\ggibson3\Documents\Manuscripts\Negative%20NRG\PLGE\12485966).

71. Cholesterol Treatment Trialists Collaborators. The effects of lowering LDL cholesterol with statin therapy in people at low risk of vascular disease: meta-analysis of individual data from 27 randomised trials. Lancet 2012; 380(9841): 581-90. PMID: [22607822](file:///C:\Users\ggibson3\Documents\Manuscripts\Negative%20NRG\PLGE\22607822).

72. Stone NJ, Robinson JG, Lichtenstein AH, Bairey Merz CN, Blum CB, Eckel RH, et al. 2013 ACC/AHA guideline on the treatment of blood cholesterol to reduce atherosclerotic cardiovascular risk in adults: a report of the American College of Cardiology/American Heart Association Task Force on Practice Guidelines. Circulation. 2014; 129(25 Suppl 2): S1-45. PMID: [24222016](https://www.ncbi.nlm.nih.gov/pubmed/?term=24222016).

73. Greving JP, Visseren FL, de Wit GA, Algra A. Statin treatment for primary prevention of vascular disease: whom to treat? Cost-effectiveness analysis. BMJ 2011; 342: d1672. PMID: [21450800](file:///C:\Users\ggibson3\Documents\Manuscripts\Negative%20NRG\PLGE\21450800).

74. Maningat P, Gordon BR, Breslow JL. How do we improve patient compliance and adherence to long-term statin therapy? Curr Atheroscler Rep. 2013; 15(1): 291. PMID: [23225173](file:///C:\Users\ggibson3\Documents\Manuscripts\Negative%20NRG\PLGE\23225173).

75. Mega JL, Stitziel NO, Smith JG, Chasman DI, Caulfield M, Devlin JJ, et al. Genetic risk, coronary heart disease events, and the clinical benefit of statin therapy: an analysis of primary and secondary prevention trials. Lancet 2017; 385: 2264-71. PMID: [25748612](https://www.ncbi.nlm.nih.gov/pubmed/?term=25748612).

76. Natarajan P, Young R, Stitziel NO, Padmanabhan S, Baber U, Mehran R, et al. Polygenic risk score identifies subgroup with higher burden of atherosclerosis and greater relative benefit from statin therapy in the primary prevention setting. Circulation 2017; 135(22): 2091-101. PMID: [28223407](https://www.ncbi.nlm.nih.gov/pubmed/?term=28223407).

77. Willcutt EG. The prevalence of DSM-IV attention-deficit/hyperactivity disorder: a meta-analytic review. Neurotherapeutics 2012; 9(3): 490-9. PMID: [22976615](file:///C:\Users\ggibson3\Documents\Manuscripts\Negative%20NRG\PLGE\22976615).

78. Catalá-López F, Hutton B, Núñez-Beltrán A, Page MJ, Ridao M, Macías Saint-Gerons D, et al. The pharmacological and non-pharmacological treatment of attention deficit hyperactivity disorder in children and adolescents: A systematic review with network meta-analyses of randomised trials. PLoS ONE 2017; 12(7): e0180355. PMID: [28700715](file:///C:\Users\ggibson3\Documents\Manuscripts\Negative%20NRG\PLGE\28700715).

79. Fredriksen M, Dahl AA, Martinsen EW, Klungsøyr O, Haavik J, Peleikis DE. Effectiveness of one-year pharmacological treatment of adult attention-deficit/hyperactivity disorder (ADHD): an open-label prospective study of time in treatment, dose, side-effects and comorbidity. Euro Neuropsychopharmacol 2014; 24(12): 1873-1884. PMID: [25453480](https://www.ncbi.nlm.nih.gov/pubmed/?term=25453480).

80. Haertling F, Mueller B, Bilke-Hentsch O. Effectiveness and safety of a long-acting, once-daily, two-phase release formulation of methylphenidate (Ritalin® LA) in school children under daily practice conditions. Atten Defic Hyperact Disord. 2015;7(2): 157-64. PMID: [25346231](https://www.ncbi.nlm.nih.gov/pubmed/?term=25346231).

81. Wang GJ, Volkow ND, Wigal T, Kollins SH, Newcorn JH, Telang F, et al. Long-term stimulant treatment affects brain dopamine transporter level in patients with attention deficit hyperactive disorder. PLoS ONE 2013; 8(5): e63023. PMID: [23696790](https://www.ncbi.nlm.nih.gov/pubmed/?term=23696790).

82. Van der Schans J, Kotsopoulos N, Hoekstra PJ, Hak E, Postma MJ. Cost-effectiveness of extended-release methylphenidate in children and adolescents with attention-deficit/hyperactivity disorder sub-optimally treated with immediate release methylphenidate. PLoS ONE 2015; 10: e0127237. PMID: [26024479](https://www.ncbi.nlm.nih.gov/pubmed/?term=26024479).

83. Faraone SV, Perlis RH, Doyle AE, Smoller JW, Goralnick JJ, Holmgren MA, Sklar P. Molecular genetics of attention-deficit/hyperactivity disorder. Biol Psychiatry. 2005; 57(11): 1313-23. PMID: [15950004](https://www.ncbi.nlm.nih.gov/pubmed/?term=15950004).

84. Demontis D, Walters RK, Martin J, Mattheisen M, Als TD, Agerbo E, et al. Discovery of the first genome-wide significant risk loci for attention deficit/hyperactivity disorder. Nat Genet. 2018; In press. PMID: [30478444](https://www.ncbi.nlm.nih.gov/pubmed/?term=30478444).

85. Stergiakouli E, Martin J, Hamshere ML, Langley K, Evans DM, St Pourcain B, et al. Shared genetic influences between attention-deficit/hyperactivity disorder (ADHD) traits in children and clinical ADHD. J Am Acad Child Adolesc Psychiatry. 2015; 54(4): 322-7. PMID: [25791149](https://www.ncbi.nlm.nih.gov/pubmed/?term=25791149).

86. Martin J, Hamshere ML, Stergiakouli E, O'Donovan MC, Thapar A. Genetic risk for attention-deficit/hyperactivity disorder contributes to neurodevelopmental traits in the general population. Biol Psychiatry. 2014; 76(8): 664-71. PMID: [24673882](https://www.ncbi.nlm.nih.gov/pubmed/?term=24673882).

87. Urban TJ, Goldstein DB. Pharmacogenetics at 50: genomic personalization comes of age. Sci Transl Med. 2014; 6(220): 220ps1. PMID: [24452261](https://www.ncbi.nlm.nih.gov/pubmed/?term=24452261).

88. Tate SK, Depondt C, Sisodiya SM, Cavalleri GL, Schorge S, Soranzo N, et al. Genetic predictors of the maximum doses patients receive during clinical use of the anti-epileptic drugs carbamazepine and phenytoin. Proc Natl Acad Sci (USA) 102: 5507-12. PMID: [15805193](https://www.ncbi.nlm.nih.gov/pubmed/?term=15805193).

89. Balestrini S, Sisodiya SM. Pharmacogenomics in epilepsy. Neurosci Letters 2018; 667: 27-39. PMID: [28082152](https://www.ncbi.nlm.nih.gov/pubmed/?term=28082152).

90. EpiPM Consortium. A roadmap for precision medicine in the epilepsies. Lancet Neurol. 2015; 14(12): 1219-28. PMID: [26416172](https://www.ncbi.nlm.nih.gov/pubmed/?term=26416172).

91. Striano P, Vari MS, Mazzocchetti C, Verrotti A, Zara F. Management of genetic epilepsies: From empirical treatment to precision medicine. Pharmacol Res. 2016; 107: 426-9. PMID: [27080588](https://www.ncbi.nlm.nih.gov/pubmed/?term=27080588).

92. Klepper J, Scheffer H, Leiendecker B, Gertsen E, Binder S, Leferink M, et al. Seizure control and acceptance of the ketogenic diet in GLUT1 deficiency syndrome: a 2- to 5-year follow-up of 15 children enrolled prospectively. Neuropediatrics. 2005; 36(5): 302-8. PMID: [16217704](https://www.ncbi.nlm.nih.gov/pubmed/?term=16217704).

93. Mills PB, Struys E, Jakobs C, Plecko B, Baxter P, Baumgartner M, et al. Mutations in antiquitin in individuals with pyridoxine-dependent seizures. Nat Med. 2006; 12(3): 307-9. PMID: [16491085](https://www.ncbi.nlm.nih.gov/pubmed/?term=16491085).

94. Brunkow ME, Gardner JC, Van Ness J, Paeper BW, Kovacevich BR, Proll S, et al. Bone dysplasia sclerosteosis results from loss of the SOST gene product, a novel cystine knot-containing protein. Am J Hum Genet. 2001; 68(3): 577-89. PMID: [11179006](https://www.ncbi.nlm.nih.gov/pubmed/?term=11179006).

95. Nishino M, Ramaiya NH, Hatabu H, Hodi FS. Monitoring immune-checkpoint blockade: response evaluation and biomarker development. Nat Rev Clin Oncol. 2017; 14(11):655-68. PMID: [28653677](https://www.ncbi.nlm.nih.gov/pubmed/?term=28653677).

96. Sutherland A, Power RJ, Rahman P, O'Rielly DD. Pharmacogenetics and pharmacogenomics in psoriasis treatment: current challenges and future prospects. Exp Op Drug Metab Tox. 2016; 12(8): 923-35. PMID: [27266955](https://www.ncbi.nlm.nih.gov/pubmed/?term=27266955).

97. Ovejero-Benito, MC, Muñoz-Aceituno E, Reolid A, Saiz-Rodríguez M, Abad-Santos F. Pharmacogenetics and pharmacogenomics in moderate-to-severe psoriasis (2017) Am J Clin Dermatol 19(2): 209-222. PMID: [28921458](https://www.ncbi.nlm.nih.gov/pubmed/?term=28921458).

98. Papp KA, Blauvelt A, Bukhalo M, Gooderham M, Krueger JG, Lacour JP, et al. Risankizumab versus Ustekinumab for moderate-to-severe plaque psoriasis. N Engl J Med. 2017; 376(16): 1551-60. PMID: [28423301](https://www.ncbi.nlm.nih.gov/pubmed/?term=28423301).

99. Rusta-Sallehy S, Gooderham M, Papp K. Brodalumab: a review of safety. Skin Therapy Lett. 2018; 23(2): 1-3. PMID: [29562088](https://www.ncbi.nlm.nih.gov/pubmed/?term=29562088).

100. Farahnik B, Beroukhim K, Abrouk M, Nakamura M, Zhu TH, Singh R, et al. Brodalumab for the treatment of psoriasis: a review of Phase III trials. Dermatol Ther (Heidelb). 2016; 6(2): 111-24. PMID: [27221323](https://www.ncbi.nlm.nih.gov/pubmed/?term=27221323).

101. Krueger JG, Fretzin S, Suárez-Fariñas M, Haslett PA, Phipps KM, Cameron GS, et al. IL-17A is essential for cell activation and inflammatory gene circuits in subjects with psoriasis. J Allergy Clin Immunol. 2012; 130(1): 145-54.e9. PMID: [22677045](https://www.ncbi.nlm.nih.gov/pubmed/?term=22677045).

102. Banchereau R, Hong S, Cantarel B, Baldwin N, Baisch J, Edens M, et al. Personalized immunomonitoring uncovers molecular networks that stratify lupus patients. Cell. 2016; 165(3): 551-65. PMID: [27040498](https://www.ncbi.nlm.nih.gov/pubmed/?term=27040498).

103. Feuerstein JD, Cheifetz AS. Crohn Disease: Epidemiology, Diagnosis, and Management. Mayo Clin Proc. 2017; 92(7): 1088-103. PMID: [28601423](https://www.ncbi.nlm.nih.gov/pubmed/?term=28601423).

104. Lichtenstein GR, Hanauer SB, Sandborn WJ; Practice Parameters Committee of American College of Gastroenterology. Management of Crohn's disease in adults. Am J Gastroenterol. 2009; 104(2): 465-83. PMID: [19174807](https://www.ncbi.nlm.nih.gov/pubmed/?term=19174807).

105. Kugathasan S, Denson LA, Walters TD, Kim MO, Marigorta UM, Schirmer M, et al. Prediction of complicated disease course for children newly diagnosed with Crohn's disease: a multicentre inception cohort study. Lancet 2017; 389(10080): 1710-8. PMID: [28259484](https://www.ncbi.nlm.nih.gov/pubmed/?term=28259484).

106. West NR, Hegazy AN, Owens BMJ, Bullers SJ, Linggi B, Buonocore S, et al. Oncostatin M drives intestinal inflammation and predicts response to tumor necrosis factor-neutralizing therapy in patients with inflammatory bowel disease. Nat Med. 2017; 23(5): 579-89. PMID: [28368383](https://www.ncbi.nlm.nih.gov/pubmed/?term=28368383).

107. Lee JC, Biasci D, Roberts R, Gearry RB, Mansfield JC, Ahmad T, et al. Genome-wide association study identifies distinct genetic contributions to prognosis and susceptibility in Crohn's disease. Nat Genet. 2017; 49(2): 262-8. PMID: [28067912](https://www.ncbi.nlm.nih.gov/pubmed/?term=28067912).

108. Vermeire S, Loftus EV Jr, Colombel JF, Feagan BG, Sandborn WJ, Sands BE, et al. Long-term efficacy of vedolizumab for Crohn's disease. J Crohns Colitis. 2017; 11(4): 412-424. PMID: [27683798](https://www.ncbi.nlm.nih.gov/pubmed/?term=27683798).
